# The Strategy to Survive Primary Malaria Infection: An Experimental Study on Behavioural Changes in Parasitized Birds

**DOI:** 10.1371/journal.pone.0159216

**Published:** 2016-07-19

**Authors:** Andrey Mukhin, Vaidas Palinauskas, Elena Platonova, Dmitry Kobylkov, Irina Vakoliuk, Gediminas Valkiūnas

**Affiliations:** 1 Biological Station Rybachy of Zoological Institute RAS, Rybachy, 238535, Russia; 2 Nature Research Centre, Akademijos 2, 08412, Vilnius, Lithuania; 3 St. Petersburg State University, St. Petersburg, 199034, Russia; 4 Institute für Biologie und Umweltwissenschaften, University of Oldenburg, Oldenburg, 26111, Germany; 5 Immanuel Kant Baltic Federal University, Kaliningrad, 236041, Russia; University of Heidelberg Medical School, GERMANY

## Abstract

Avian malaria parasites (Haemosporida, *Plasmodium*) are of cosmopolitan distribution, and they have a significant impact on vertebrate host fitness. Experimental studies show that high parasitemia often develops during primary malaria infections. However, field studies only occasionally reveal high parasitemia in free-living birds sampled using the traditional methods of mist-netting or trapping, and light chronic infections predominate. The reason for this discrepancy between field observation and experimental data remains insufficiently understood. Since mist-netting is a passive capture method, two main parameters determine its success in sampling infected birds in wildlife, i. e. the presence of parasitized birds at a study site and their mobility. In other words, the trapping probability depends on the survival rate of birds and their locomotor activity during infection. Here we test (1) the mortality rate of wild birds infected with *Plasmodium relictum* (the lineage pSGS1), (2) the changes in their behaviour during presence of an aerial predator, and (3) the changes in their locomotor activity at the stage of high primary parasitemia.We show that some behavioural features which might affect a bird's survival during a predator attack (time of reaction, speed of flush flight and take off angle) did not change significantly during primary infection. However, the locomotor activity of infected birds was almost halved compared to control (non-infected) birds during the peak of parasitemia. We report (1) the markedly reduced mobility and (2) the 20% mortality rate caused by *P*. *relictum* and conclude that these factors are responsible for the underrepresentation of birds in mist nets and traps during the stage of high primary parasitemia in wildlife. This study indicates that the widespread parasite, *P*. *relictum* (pSGS1) influences the behaviour of birds during primary parasitemia. Experimental studies combined with field observations are needed to better understand the mechanisms of pathogenicity of avian malaria parasites and their influence on bird populations.

## Introduction

The evidence for parasite impacts on their hosts has been reported in various host-parasite associations [1]. The influence of parasites on their hosts is manifold, ranging from simple exploitation of the existing host resources to complicated host behavioural alterations that facilitate parasite transmission [2]. Manipulation of host behaviour by pathogens might have detrimental consequences for entire host populations due to the alterations of surrounding animal communities and the influence on host evolution and speciation [2–5].

Avian haemosporidian parasites (Apicomplexa, Haemosporida) belonging to the genera *Leucocytozoon*, *Haemoproteus* and also the agents of malaria, i. e. *Plasmodium* spp., are prevalent worldwide, and they infect the great majority of terrestrial avian species, particularly birds belonging to Passeriformes [6]. Up to 80% morbidity due to haemosporidian infections has been reported in some bird populations [6]. However, light (chronic) parasitemia of *Plasmodium* spp. predominates in the great majority of wild-caught birds [6,7]. The high prevalence of haemosporidians, combined with the predominantly light chronic parasitemia in some bird species has led to the conclusion that these blood parasites are relatively benign in their avian hosts [8]. However, according to the literature reviewed by Valkiūnas and Atkinson et al. [6,9] and also recent experimental studies [10–14], one of the most critical periods for the survival of malaria-infected hosts is the primary infection stage, which is difficult to assess during field studies. During primary infection, *Plasmodium* spp. parasites undergo rapid asexual multiplication in red blood cells resulting in acute anaemia. Additionally, exoerythrocytic meronts of malaria parasites cause massive damage of tissues in various organs [15,16]. The harm caused by *Plasmodium* parasites has been well documented in naturally infected non-adapted exotic birds [17–19]. Several recent studies revealed that some species of haemosporidian parasites are dangerous and cause negative fitness consequences or even death of birds [12,15,20–23]. In recent study[15] wild bird species that were found dead in Vienna and other sites in eastern Austria were examined. The birds revealed a massive infestation of exoerythrocytic meronts of *Plasmodium* parasites in various organs including the brain, spleen and lungs. The majority of cited studies or case reports have shown that avian haemosporidian parasites can be dangerous not only for non-adapted bird species like, for example, penguins [24] or endemic Hawaiian honeycreepers of the Drepaniidae [9] that did not co-evolve with these parasites, but also in bird species, which have been evolutionarily exposed to these infections for long periods, for example, the blackbird *Turdus merula* in Europe [15]. Based on limited available experimental data and scarce case reports from wildlife, it is difficult to estimate how many individuals from certain populations get malaria infections, how many of them survive, and what are the behavioural changes in the parasitized birds. This information is crucial for determining factors that are important for the survival of infected birds in nature. Answering these questions would contribute to the better understanding of host-parasite interactions and epidemiology of avian malaria in wildlife.

A prominent obstacle in evaluating the true impact of haemosporidians and other parasites on wild birds is the methodology of sampling in wildlife [25]. Mist-netting or trapping of birds are common methods to collect blood samples and to assess the number of infected individuals in different populations [6,7,26,27]. Being easy to use, mist-netting and trapping however might provide biased data about the number of hosts at the stage of acute malaria infection in a population. Unlike experimental studies, in which the primary parasitemia of *Plasmodium* spp. often exceeds 10% [10–12,14,16], a parasitemia intensity of > 1% is almost never observed in samples collected from mist-netted birds [20,28–31]. This discrepancy was reported by Valkiūnas [6] who determined haemosporidian parasitemia intensity in the same population of juvenile chaffinches *Fringilla coelebs* that were mist-netted and shot in parallel on the Curonian Spit in the Baltic Sea. Heavy parasitemia was reported in some individuals of shot chaffinches, but not in the mist-netted individuals. The reasons for this might be the decrease of the birds’ locomotor activity resulting in passive behaviour of individuals at the stage of high parasitemia and the higher predation pressure on the infected individuals, or both. Due to this shortcoming of the widely used methods, the available data about common light natural infections and harmlessness of malaria and related parasites in wild birds should be interpreted with caution, particularly in regard of impact of these parasites on host fitness.

In the present study we investigated the development and impact of behaviour of the worldwide-distributed avian malaria parasite *Plasmodium relictum* (lineage pSGS1) in experimentally infected siskins (*Carduelis spinus*). We followed the development of primary parasitemia in the experimentally infected siskins and measure this infection impact on (1) the reaction of birds to the presence of an aerial predator and (2) the overall locomotor activity of control and parasitized individuals. *Plasmodium relictum* (pSGS1) was used in our experiments because it is the most prevalent malaria parasite infecting 106 bird species around the world (according to the MalAvi database on 15.10.2015, see http://mbio-serv2.mbioekol.lu.se/Malavi/index.html, [32]). This infection is actively transmitted up to the Arctic Circle [33,34], and it affects birds with varying behavioural ecologies in various habitats.

## Material and Methods

### Study Site

Data about the prevalence and intensity of haemosporidian infections were obtained from trapped birds during spring—summer seasons in 2011–2013. All birds were caught using mist-nets and big funnel traps [35] at the Biological Station Rybachy, Kaliningrad region, Russia (54°57'N, 20°31'E). In total, 1854 passerine birds belonging to 57 species were caught and examined for haemosporidian parasites using microscopy (see below).

The experimental study was carried out in June–July 2013. In all, 20 juvenile siskins were trapped during their post-fledging movements and used in our experiments. All wild-caught siskins were proved to be free of haemosporidian parasites using both microscopic and polymerase chain reaction (PCR)-based methods (see below).

### Housing

After a week of adaptation to captivity, siskins were randomly split into control and experimental groups (10 birds in each group). Each bird was placed into an individual plastic cage (60×40×40 cm, Joko GmbH, Germany). All cages were housed in a vector-free laboratory under controlled temperature (20°C) and photoperiod (LD 17:7), which was maintained by using individual luminous tube lamps placed over each cage. All birds received water and food *ad libitum*.

### Infection Procedures

For experimental infection, *P*. *relictum* (mitochondrial cytochrome *b* gene lineage pSGS1, GenBank accession no. JX993045) was used. The parasite was originally isolated from a naturally infected red crossbill (*Loxia curvirostra*) in 2010. This strain was multiplied and cryopreserved at the Nature Research Centre, Lithuania [16]. One sample of frozen blood was thawed, multiplied by infection of one uninfected red crossbill and one siskin, and used to infect experimental siskins, as described by Palinauskas et al. [16]. The seventh passage of the original isolate was used in this study.

About 0.15 ml of freshly prepared mixture of infected blood, 3.7% sodium citrate and 0.9% saline (4:1:5) was used to expose experimental siskins by subinoculation of the mixture in the pectoral muscles, as described by Palinauskas et al. [11]. Intensity of mature meronts in the blood was 0.075%, and the dose of inoculated meronts in each bird was approximately 2 × 10^5^. Ten siskins were used as negative controls, and they were exposed to the same amount of non-infected blood mixture obtained from a red crossbill.

### Microscopic Examination and PCR-Based Analysis

After infection of the experimental group (0 day), parasitemia was determined every fourth day up to 36 day post inoculation (dpi). Blood was taken by puncturing the brachial vein using sterile syringe needle, collected in heparinized capillaries and used to prepare two blood films. About 20 μl of the blood also was stored in SET-buffer [36] for molecular analysis, and 40 μl was used for haematocrit measurements (presented elsewhere).

An Olympus CH2O light microscope was used to examine slides. Approximately 100 fields were studied at high magnification (1000×). Intensity of parasitemia was estimated as a percentage by actual counting of the number of parasites per 1000 erythrocytes or per 10,000 erythrocytes if infections were light (<0.1%), as recommended by Godfrey et al. [37].

PCR-based analysis was performed according to [16]. Briefly, total DNA was extracted from blood using an ammonium acetate protocol [38]. Extracted DNA was used as a template for nested PCR [36]. To amplify 479 bp fragment of the mitochondrial cytochrome *b* gene, we used the primers HaemFNI/HaemNR3 and HAEMF/HAEMR2 [36,39]. Thermal conditions for DNA amplifications were the same as defined by Hellgren et al. [36]. The amplification success was evaluated by running the PCR product (1.5μl) on 2% agarose gel. Positive samples were sequenced from the 5’ end with the primer HAEMF as described by Bensch et al. [39]. A negative control (nuclease-free water) was used every 7 samples to control for false amplifications. No case of false amplification was found.

### Experiment Design and Behavioural Tests

**Fig 1:**
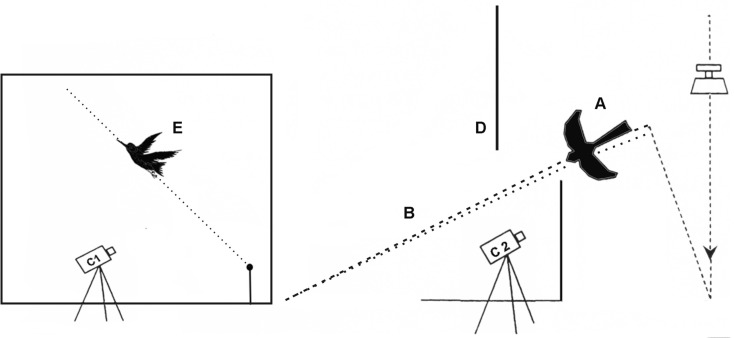
Behavioural setup. Behavioural setup with a predator dummy (A, *Falco columbarius*) speeding down along a fishing line (B). Two video cameras (C1 and C2) film the appearence of the predator dummy behind a shield (D) and the resulting take-off of an attacked experimental bird (E).

Behavioural characteristics both of experimental and control bird groups were tested using an aerial predator dummy according to the [40] setup, which was slightly modified during this study. This setup represents the aerial predator dummy of Merlin (*Falco columbarius*) rapidly sliding down along a fishing line under a force of a fallen weight (Fig 1B). We used a fallen weight (5 kg) to speed movement of the dummy (Fig 1A). The dummy was released from height of approximately 2.5 m. The plastic dummy of falcon shape "attacked" the cage (160×44×65 cm) containing single perch located near a feeder which usually was occupied by a tested siskin. Two synchronized video cameras permanently recorded 1) the appearance of the dummy from beyond the shield and 2) the bird's escape take-off activity (Fig 1).

The entire setup was constructed in a separate room. First, each tested bird was placed into this setup for one hour (an adaptation period). To prevent birds’ responses to noises made by the sliding dummy and the fallen weight, we applied constant recording of sham noise during this experiment. Then, after approximately one hour of the adaptation and when a bird was sitting on the perch, the dummy was manually released by an observer. Video recordings of all tests were analysed by the drawing program as series of video snapshots.

The following characteristics of bird escape behaviour were measured: (1) time of reaction (the time, which took to realise that bird is under attack and to response to appearance of the dummy by escape flight); (2) angle of take-off flight; (3) speed of bird escape flight.

We tested all birds twice under these conditions. The first trial was done before experimental infections of birds and the second trial was done when maximum parasitemia was reached in the experimental birds.

### Recording of the Locomotor Activity

All cages were equipped with an infrared detector (S-230A, Conrad Electronic GmbH, Germany) attached to a front site of all cages. These detectors recorded birds' locomotor activity continuously during the whole experiment. Our recorded system summarized the number of jumps per 5 min intervals and stored this information in a computer database.

### Statistical Analysis

All statistical analyses were performed using R-statistics [41].

Fuel loads (FL) was calculated as FL = (M–M_0_)/M_0_*100%, where M–birds mass on measurement, M_0_ –lean body mass. Lean body mass was estimated from a regression model of body mass on wing length using trapped siskins with a fat score of zero. Subcutaneous fat was scored from 0 to 8 after [42].

#### Escape from the predator attack

We used three measurements as indicators of changes in behaviour of infected and control birds in response to frightening by the predator model (dummy): (1) time of reaction, (2) speed of flight and (3) angle of take-off. As explanatory variables we selected the following factors: (1) group (experimental/control), (2) test (first/second), and (3) FL (fuel loads, % of lean body mass). In the models, where “speed of flight” and “angle of take-off” were used as response variables, the explanatory variable “wing length” was added. Since our data represent a repeated measurements design and they do not show violation of normality assumptions, we used linear mixed effect models for analysis with a factor “individual” as a random factor. As parasite infection might influence not only birds’ escape behaviour characteristics, but also their overall fitness, we developed an additional model, where “FL” was used as a response variable and “group” and “test” were used as explanatory variables.

First, we selected optimal random structure (random-intercept model) for a “beyond optimal model” (the model with all explanatory variables and their interactions) according to a distribution of residuals. If heteroscedasticity in residuals between groups was detected using the Fligner-Killeen Test and visual assessment of residuals’ distribution, we modified our model and added a weight parameter for corresponding variances. After we found the optimal residual variance structure using Restricted Maximum Likelihood (REML), we reduced progressively the “beyond optimal” model using Maximum Likelihood estimation method and backward model selection (selecting models with the lowest AIC) until the most parsimonious model was estimated (package “MASS”, function step AIC). The parameters of this optimal model were estimated using the restricted Maximum Likelihood method. We applied F-statistic to obtain significance levels for each factor, with a p-level of <0.05 considered to be significant. As “ANOVA” function applies sequential testing, the significance level can depend on the order of factors within the model, so we changed the order of factors and represented only consistent significant results.

For analyses of locomotor activity we used a total number of registered jumps per day. The entire course of experiment was divided into three periods based on comparing the day by day activity of control birds with experimental ones (Student’s t-test was used). As a beginning of the most severe period (second period) of parasitemia, we selected the first day of observation when the bird's activity started to be significantly different between experimental and control groups. Vice versa, the last day, when activity differed between control and experimental groups was chosen to be the last day of this period. The mean differences in activity were calculated between experimental and control groups for each period.

### Ethical Statement

The experiments described herein comply with the current laws of Russia and Lithuania. All experimental procedures were according to All Union State standard (ГОСТ № Р53434–2009 "Principles of good laboratory practice") of Russian Federation and State Food and Veterinary Service Requirements on the protection of animals used for scientific purposes (No. B1-866, 2012/10/31) of Republic of Lithuania.

All experimental procedures were approved by Specialized Committee of the Scientific Council of the Zoological Institute RAS (Saint Petersburg, Russia) and International Research Co-operation Agreement between the Biological Station Rybachy of the Zoological Institute of the Russian Academy of Sciences and Institute of Ecology of Nature Research Centre (Vilnius, Lithuania) (№ 25-05-2010).

All culling of experimental birds were permitted by Forest and Nature Protection Agency of Kaliningrad Region, Russia (№ 3-03-2010) whose permits based on the decisions made by Specialized Committee of the Scientific Council of the Zoological Institute RAS and Russian Foundation for Basic Research.

All efforts were made to minimize handling time and potential suffering of birds. To assess the health of the animals trained professional monitored each bird daily by checking if they have normal disposition, are able to keep the balance and have smooth respiratory, wings are held close to the body and if they are not sitting with tucked head. Birds were regularly weighed at the same time. Human endpoint had not been used in our experiment as infected birds did not show any sign of heavy suffering. Two cases of death occurred spontaneously and were recorded in the morning. All other experimental birds with decreased activity recovered to full health. Control birds were released after experiment; all experimental animals were euthanized by decapitation as recommended in Directive of the European Parliament and of the Council on the Protection of animals used for scientific purposes PE-CONS 37/10.

## Results

### Parasitemia in Wild-Caught Birds at the Study Site

As an indication of parasite prevalence in the community: in all, 753 trapped birds were infected with haemosporidians (*Haemoproteus* spp., *Leucocytozoon* spp.), and 76 of them were infected with *Plasmodium* spp. Parasitemia was of ≤0.1% in all infected birds, except 4 individuals. In three of these individuals, *Plasmodium* spp. parasitemia ranged between 1 and 2%, and one bird had parasitemia about 3%.

### Parasitemia and Bird Survival of Experimentally Infected Birds

According to the PCR-based analysis and microscopic examination of blood samples, the control birds remained non-infected through the course of experiment.

**Fig 2:**
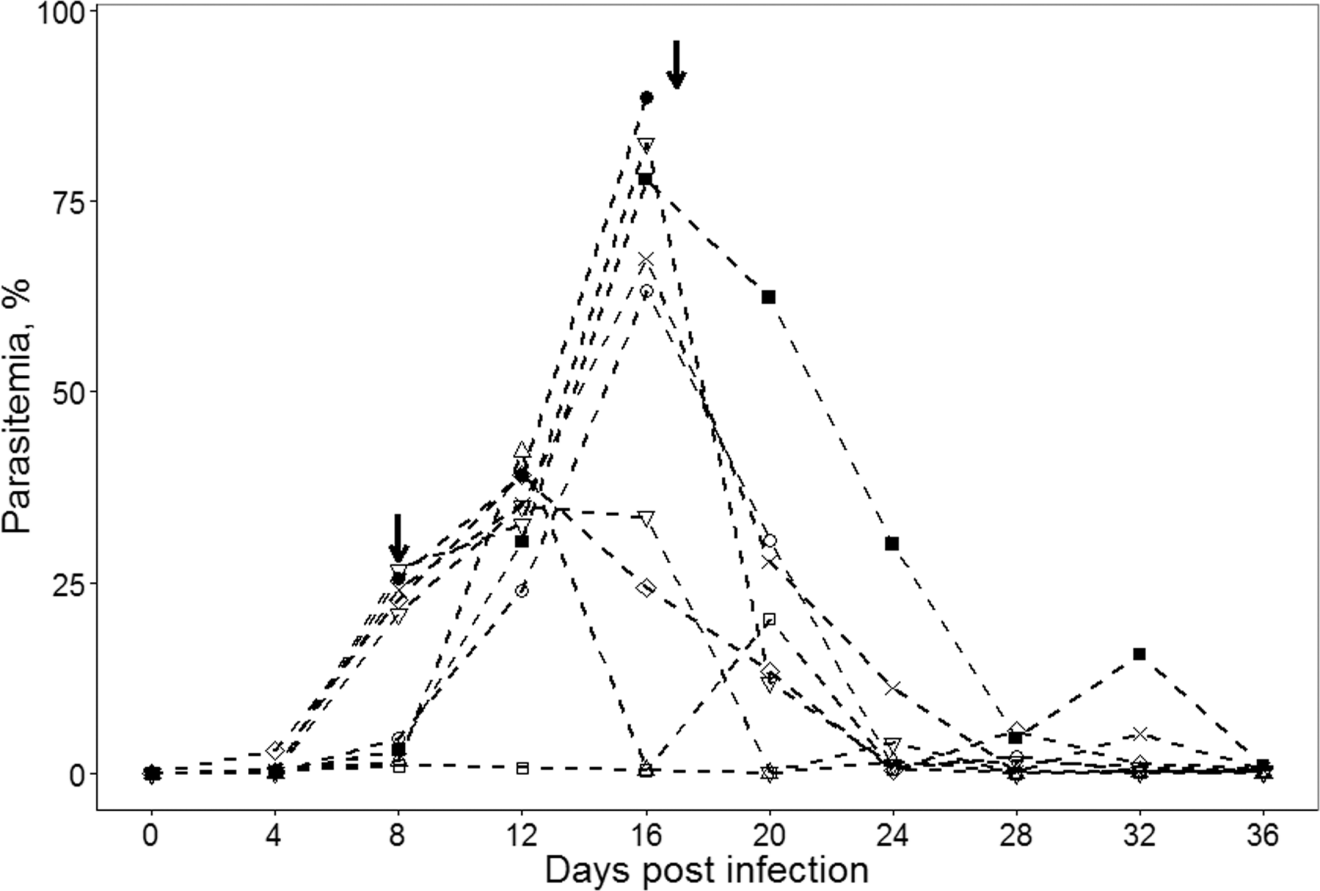
Dynamics of parasitemia. Dynamics of parasitemia of *Plasmodium relictum* (lineage pSGS1) in different individuals of experimentally infected siskins. Symbols represent different individuals; arrows mark birds dead on 8 and 17 dpi.

All experimentally infected birds were susceptible to *P*. *relictum*. PCR analysis confirmed the presence of pSGS1 lineage in all experimentally infected birds. The prepatent period was 4 dpi. On 4 dpi, parasitemia (n = 10) was (Limit, Mean±SD) 0.1–3.1, 0.5±0.9%. Maximum parasitemia (n = 9) was reported 16 dpi; it was 26.8–88.5, 55.4±20.9%. On 36 dpi (n = 8), parasitemia was 0.0–1.0, 0.3±0.4%, and could be considered at the chronic stage of infection (Fig 2).

Mortality was not reported among birds of the control group. Two birds (20%) from the experimental group died. One bird died 8 dpi with parasitemia of 3.0%, and the second one died 17 dpi with approximately 88% parasitemia.

### Escape from the Predator Attack

Three birds with parasitemia of 3%, 88.5% and 42.2% from experimental group did not show any escape flight during the second trial test. They were entirely apathetic to the approaching dummy. We excluded these birds from statistical analysis without any fictitious results (for instance, like more than 1 sec), which would definitely increase the differences between the groups. Such birds were not reported in control group.

Results of LME presented in Table 1.

**Table 1:** Linear mixed effect models.

Response variable	Explanatory variable	Figures
Time of reaction	test* + FL	Fig 3A
Angle of take-off	test + group + FL* + wing + test: FL + test: group + test: wing + group: wing + test: group: FL*	Fig 3B
Speed of flight	test* + group + FL + wing* + test: group + test: wing* + group: FL + group: wing + FL: wing* + test: group: wing	Fig 3C

**Fig 3:**
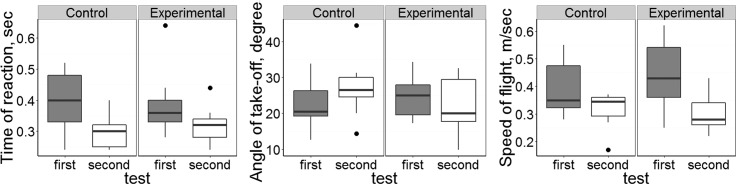
Changes in behavioural characteristics. Changes of time reaction, angle of take-off, and speed of flight in control (non-infected) and *Plasmodium relictum* (lineage pSGS1) infected birds. Boxes and whiskers represent the upper/lower qartiles ± maximum/minimum values of distribution: grey boxes—first test before experiment, white boxes–second test after exposure. Points indicate outliers, solid horizontal lines show medians.

LME models used in the statistical analysis of experimental data. Factors with significant effect (F-statistics, p<0.05) are marked with asterisks (*). Fig 3 represents the influence of the main interest factor (test: group). Note. Only optimal models are presented.

1. “Time of reaction”.

The optimal model after the backward selection was: “time of reaction” ~ “test” + “FL”. Although time of reaction indeed decreased from the first test to the second one (see Fig 3, p = 0.0045), parasitemia itself does not alter the reaction of birds because this effect was evident in both experimental and control groups. In the second trial, birds from both groups responded slightly quicker to the predator, but flew up from the perch with less speed (Table 1).

Although factor “FL” was included in the optimal model, its effect was not significant (p = 0.098).

2. “Angle of take-off”.

The optimal model for take-off angle after the backward selection is: “Angle”~ “test” + “group” + “FL” + “wing” + “test: FL” + “test: group” + “test: wing” + “group: wing” + “test: group: FL”

**Fig 4:**
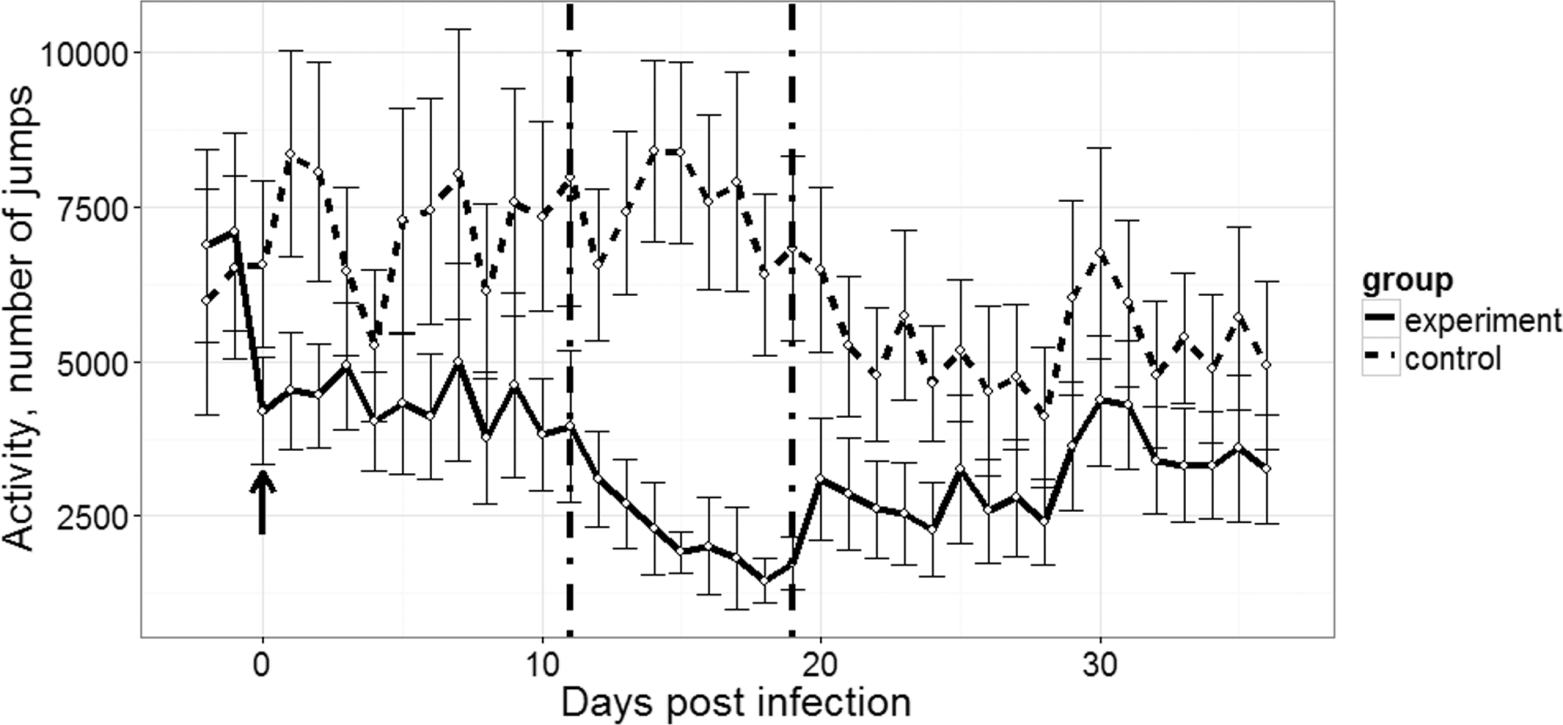
Changes in locomotor activity. Locomotor activity as a mean number of jumps per day (±SEM) of experimentally infected with *Plasmodium relictum* (lineage pSGS1) (solid line) and non-infected (control, dashed line) groups of siskins. Arrow shows the day of exposure. Vertical dash-dot lines set bounds to the most severe stage of parasitemia.

Optimal model for the variable “angle” looks rather complicated and includes all explanatory variables and also some interactions between them. Only two of them significantly influenced the response variable, irrespective of the order of factors in the model: “FL” (p = 0.01), and “test: group: FL” (p = 0.02) (Figs 3 and 4). The effect of the main-interest factor “test: group” was on the limit to significant (p = 0.07) and p-level depended on the sequence of entrance.

3. “Speed of flight”.

The optimal model after the backward selection is: “Speed of flight” ~ “test” + “group” + “FL” + “wing” + “test: group” + “test: wing” + “group: FL” + “group: wing” + “FL: wing” + “test: group: wing”

Optimal models were selected according to the Akaike Information Criterion. Explanatory variables: “group” (experimental/control), “test” (first/second), and “FL” (fuel loads, % of lean body mass). In models, in which “Speed of flight” and “Angle of take-off” parameters were used as response variables, the explanatory variable “wing” (wing length) was added.

Fuel load and wing length may play an essential role during the escape flight: birds with longer wings had higher speed of flight and birds with higher body mass flew up with a lesser angle. The latter does not hold for experimental birds after infection: correlation between “fuel loads” and the angle of flight was reversed–infected birds with higher body mass flew up with greater angle.

The model with a “fuel load” as a response variable showed that the body mass itself decreases between the first and the second test (p = 0.053), but this happened both in experimental and control groups. Infection did not lead to weight loss (mean weight of birds after infection was 9.8% (experimental group) and 9.3% (control) of lean body mass, t-test p = 0.53).

According to this analysis, the infection itself did not affect the birds’ take off performance in our experiment because factor “test: group” was not significant.

### Changes in Locomotor Activity

We divided the course of parasitemia into three periods (day 0 –day 11, day 12 –day 19, and day 20 –day 36). Experimental birds substantially lowered activity already during the first days post infection. Activity of birds differed significantly between experimental and control groups during all these periods. Even during the third period, when parasitemia substantially decreased, birds did not rehabilitate fully and were rather inactive (number of jumps per day differed significantly in comparison to controls, p = 4.847e-08).

The activity of experimental birds was on average twice as low as of control birds (48% less; 3309±158 vs. 6413±242 jumps per day subsequently) during the experiment (Fig 4).

## Discussion

This is the first experimental study testing the influence of avian malaria parasites on wild birds during high primary parasitemia using controlled laboratory behaviour tests. The key result is that *P*. *relictum* (pSGS1), a widespread agent of avian malaria, markedly reduced the locomotor activity of infected individuals. These results bring into question the accuracy of prevalence data using the traditional methods of bird mist-netting and trapping, particularly during peak of parasitemia. These data support a former conclusion, which was made using *Haemoproteus* spp. model organisms (sister group to *Plasmodium*), that heavily infected birds are underrepresented in field studies simply due to methodological shortcomings in assessing parasitized individuals in wildlife [6]. In our 3 year project of mist-netting and trapping birds at the study site, light parasitemia of haemosporidian parasites predominated, and only one individual out of 1854 examined showed parasitemia of approximately 3%. Here we show that this is likely a result of bias of sampling methodology of wild birds, and suggest that bird populations are affected by malaria more often and more severely under natural conditions.

*Plasmodium relictum* (pSGS1) developed in all experimentally infected siskins. The primary parasitemia reached the peak up to 88.5% 16 dpi. Two infected birds died during the course of infection 8 and 17 dpi. One individual likely died due to extremely high parasitemia (88.5%), which was reported just before the death. It is probable that there is a threshold level of parasitemia which bird immune system cannot resist. Direct destruction of erythrocytes, removal of infected blood cells from the circulation and the resulting anaemia likely are important reasons of mortality [12,16,23]. The death of one bird with parasitemia of 3% could be due to the damage caused by the parasite tissue stages as was the case in birds infected with *Plasmodium homocircumflexum* during light parasitemia (<1%) [16].

From an evolutionary perspective of vector-borne diseases, it should be disadvantageous for parasites to kill or weaken their hosts making them easier accessible prey for predators. The development of high parasitemia during primary infection should be the result of trade-off between the survival both of parasite and vertebrate host. Mechanisms of such host-parasite interactions remain insufficiently investigated in wildlife [9]. It might be that (1) high parasitemia is beneficial for avian malaria transmission due to development of large number of asexual forms and some gametocytes, which are necessary for vector infection and (2) the weakening of host immune system (as a factor allowing marked parasite multiplication) is the cost of maintenance of parasite in bird populations. Additional experimental studies are needed to better understand this issue. It is important to note that our experimental birds were kept in optimal laboratory conditions with constant temperature and *ad libitum* food and water supply. In wildlife, the mortality rate of infected birds is expected to be higher because of selection caused by unfavourable weather conditions, shortage of food, inter- and intra-species competition and presence of predators. We believe that the mortality rate in our controlled study was caused by the adverse effects of *P*. *relictum* infection, and not to the other environmental and ecological factors indicated above.

Testing of experimentally infected and control birds showed the same results in our escape behaviour experiments. There were no differences discernable between these two groups in bird reaction to the appearance of predator, flight speed or flight angle. However, it is important to note that 3 birds from the experimental group did not show any escape behaviour. During a true predator attack, they certainly would not have survived. Two of these birds died afterwards (same and next day after trial), but one individual survived until the end of the experiment. These data show that not only birds at the terminal stage of disease (who die in any case) but also those that otherwise would survive peak parasitemia, can be eliminated by predators. At peak parasitemia, seven infected birds showed the same responses in escape flight as the healthy (control) ones. This indicates that even heavily infected siskins can successfully escape predator attacks by short flush flights, which should be an important strategy to escape predators in wildlife. Rapid escape behaviour could be maintained by anaerobic metabolism, which does not require oxygen [43]. Transport of the latter would be interrupted due to the massive destruction of red blood cells in malaria infected animals [6,44]. For example, [45] showed that burst speed does not depend on parasitemia in naturally *Plasmodium mexicanum* infected lizards, and the parasitized animals retain a capacity for the rapid escape behaviour. However, malaria infection induced a decline in aerobic scope and was accompanied by a decline in stamina of parasitized lizards. In the present study, we applied behavioural tests, which were performed in limited size cages, thus we did not have an opportunity to test if the reported successful short escape flight would be enough to survive a predator attack in wildlife.

Recently, the study [46] showed that haemosporidian infected common nightingales (*Luscinia megarhynchos*), show higher risk-taking behaviour as compared to non-infected (control) birds, and this could result in an increase in their predation risk. On the other hand, there were no differences in exploration of a new environments between infected and control birds. The study [47] used other behaviour variables (tonic immobility and biting score) and concluded that *Plasmodium* spp. infected house sparrows were more aggressive and demonstrated more expressed escape behaviour than controls, thus theoretically the likelihood to escape from predator should increase in the former group. It is important to note that both these studies were made using birds with natural chronic infections, which already passed the most dangerous primary infection stage. We show that escape behaviour (reaction to appearance of a predator) does not change during primary parasitemia (Fig 3), but infected birds tend to have slower reaction than control ones: however, the difference was insignificant. Differences between afore-mentioned studies and our results could be due to different experimental tests and different parasite and bird species used, but more likely are due to differences in the stage of infection in tested birds. This study clarifies the effect of parasites on hosts during the primary parasitemia stage, when [46,47] studies were dealing with wild-caught birds at light (chronic) parasitemia stage. In other words, relatively healthy individuals were used in these two experimental studies, but the influence of heavy primary parasitemia on the tested bird behaviour parameters remained lacking.

In this study, the changes in locomotor activity of infected birds were directly related to the stage of parasitemia, and they were marked during high primary parasitemia (see Figs 2 and 3). We show that the increase of parasitemia is accompanied by marked decrease of activity of experimental birds in comparison to the controls. In other words, the heavily infected birds were secretive and less mobile. Such behaviour indicates disease, which is likely due to the direct damage of erythrocytes and their removal in spleen and liver. The latter organs are markedly enlarged and assume dark colour in malaria infected birds [6,9,11]. Results of this study are in agreement with [48] study, which showed that vector-borne blood parasites tend to make birds less mobile. These authors provided evidence that willow ptarmigans (*Lagopus lagopus*) infected with avian haemosporidian parasites more often choose freezing behaviour instead of fleeing, after frightening. The similar secretive behaviour was reported in juvenile chaffinches naturally infected with *Haemoproteus* parasites [6]. Additionally, heavy haemosporidian parasitemia was reported to be associated with lethargy and anorexia in snowy owls (*Bubo scandiacus*) and some passeriform birds [11,49]. The same is true in many species of captive penguins infected with *P*. *relictum* in zoos [6,24].

It is important to note that avian malaria causes conspicuous damage in many organs of birds due to the development of tissue stages, which is not the case with malaria parasites of humans and other mammals [9,44]. Bird mortality due to *Plasmodium* infection often is not only due to the blood pathology, but also damage caused by secondary erythrocytic meronts (phanerozoites), which develop in reticular cells, including epithelial cells of blood vessels in various organs and tissues [6,50–52]. Phanerozoites often develop during chronic infections, and even single phanerozoites can cause the death of avian hosts due to blockage of the circulation in capillaries of the brain, which can lead to cerebral paralysis [16]. The data on mortality caused by afflicted tissue changes are impossible to obtain if only blood samples are collected using traditional methods of capture (mist-netting, trapping). However, both these approaches predominate as methodologies in current haemosporidian studies. Further experiments are needed to clarify the importance of internal organ pathologies and the associated mortalities, which seems to be more common in malaria infected wild birds than previously thought, but remain insufficiently investigated [15].

The duration of the severe stage of the primary parasitemia of *Plasmodium* spp. is about 1–2 weeks in many species of avian malaria parasites [6,11,14,28,53]. This study shows that the mobility of infected birds drops to about half of the controls, and that this effect is particularly pronounced during peak of parasitemia (Fig 4). This period of life is crucial for survival of infected birds in wildlife because the decrease of activity is not only accompanied by disease and mortality due to malaria, but also an increase in risk to environmental and ecological pressures. Because of the mortality and reduced activity of diseased individuals during primary infections, the probability to catch birds at high parasitemia stage is reduced using mistnets or traps. Our study provides experimental evidence supporting former observations about the pathogenicity of avian malaria parasites [6,9,50].

In conclusion, the results of this experimental study show that the application of passive methods of animal catching provides only partial information about host-parasite relationships in wildlife. Data collected solely using such methods should be interpreted with caution in malaria research and probably other parasitology studies dealing with pathogenicity of infections. This is particularly true in research aimed at understanding mechanisms of pathogen impacts on vertebrate hosts. Underestimation of parasite burden, which often is short-term but renders birds vulnerable, might mislead our understanding of the true pathogenicity and disease epidemiology in wildlife. That should be taken into consideration in parasite virulence and demography studies when estimating mortality rates in wildlife populations.
